# An Overview of the Conventional and Novel Methods Employed for SARS-CoV-2 Neutralizing Antibody Measurement

**DOI:** 10.3390/v15071504

**Published:** 2023-07-05

**Authors:** Vinícius Pinto Costa Rocha, Helenita Costa Quadros, Antônio Márcio Santana Fernandes, Luana Pereira Gonçalves, Roberto José da Silva Badaró, Milena Botelho Pereira Soares, Bruna Aparecida Souza Machado

**Affiliations:** 1 Institute of Health Technology, National Industrial Learning Service—Integrated Manufacturing and Technology Campus, SENAI CIMATEC, Salvador 41650-010, Bahia, Brazil; antonio.fernandes@fbter.org.br (A.M.S.F.); luanapereira84@gmail.com (L.P.G.); badaro@fieb.org.br (R.J.d.S.B.); milena.soares@fieb.org.br (M.B.P.S.); 2 Laboratory of Tissue Engineering and Immunopharmacology, Oswaldo Cruz Foundation, Gonçalo Moniz Institute—Fiocruz, Salvador 40296-710, Bahia, Brazil; helenita_quadros@hotmail.com

**Keywords:** neutralization test, neutralizing antibodies, COVID-19, SARS-CoV-2

## Abstract

SARS-CoV-2 is the etiological agent of the coronavirus disease-19 (COVID-19) and is responsible for the pandemic that started in 2020. The virus enters the host cell through the interaction of its spike glycoprotein with the angiotensin converting enzyme-2 (ACE2) on the host cell’s surface. Antibodies present an important role during the infection and pathogenesis due to many reasons, including the neutralization of viruses by binding to different spike epitopes. Therefore, measuring the neutralizing antibody titers in the whole population is important for COVID-19’s epidemiology. Different methods are described in the literature, and some have been used to validate the main vaccines used worldwide. In this review, we discuss the main methods used to quantify neutralizing antibody titers, their advantages and limitations, as well as new approaches to determineACE2/spike blockage by antibodies.

## 1. Introduction

The severe acute respiratory syndrome coronavirus 2 (SARS-CoV-2) emerged in 2019 in an outbreak in Wuhan, the capital city of the Hubei Province of China, and is the etiological agent of the coronavirus disease (COVID-19). Since the onset of the COVID-19 pandemic, millions of infection cases and deaths have been reported due to the fast spread of the virus. The last update of the World Health Organization (WHO) revealed that more than 766 million infection cases and almost 7 million deaths have been caused by SARS-CoV-2 around the world [1].

To begin the viral infection and trigger COVID-19 pathogenesis, SARS-CoV-2 enters the host cells through the binding of its spike protein to the angiotensin-converting enzyme 2 receptors (ACE2) on the target cells [2]. During this process, to fuse virus and host cell membranes, the receptor binding domain (RBD) of the SARS-CoV-2 spike must be proteolytically activated at the S1/S2 boundary, and for that, cellular transmembrane protease serine 2 (TMPRSS2) and lysosomal cathepsin proteases perform the dissociation of the S1 and S2 subunits [3].

Antibodies have several key functions during viral infections, including complement recruitment, opsonization, constant fragment (Fc)-mediated effector functions, such as antibody-dependent cell-mediated cytotoxicity (ADCC) and antibody-dependent cellular phagocytosis (ADCP) and neutralization of the virus’ entrance [4]. Immunity against SARS-CoV-2 variants is induced through either natural infection or vaccination, with the latter being extremely important to control the pandemic [5]. The immunity generated by natural infection and vaccination are effective ways to reduce the risk of clinically severe outcomes [6,7]. In both scenarios, the production of neutralizing antibodies (NAbs) is considered a determinant factor for predicting protective immunity against SARS-CoV-2 [8,9]. Indeed, preclinical studies using NAbs as a treatment for SARS-CoV-2 infection in rhesus macaques and hamster models have shown marked reductions in viral loads in the upper and lower respiratory tracts, and also a decrease in virus-induced pathological sequelae [10].

**Figure 1:**
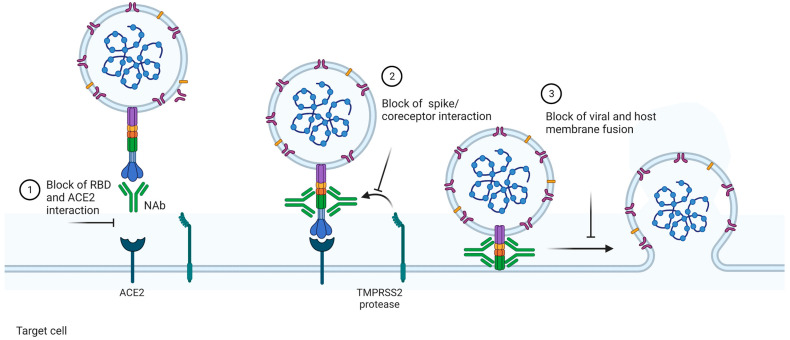
Mechanisms of action of neutralizing antibodies (NAbs). NAbs can act via different mechanisms to neutralize viruses. In this example, we focus on the neutralizing mechanisms involving SARS-CoV-2. (1) NAbs can bind to the receptor binding domain (RBD) of the spike glycoprotein from the viruses and block the contact between it and the ACE2 expressed in the surface of the host cells. Since the viral entry is dependent on this ligation, the infection is blocked. NAbs can also recognize epitopes outside the RBD. In this case, the contact between the spike and ACE2 is not avoided. However, the following mechanisms essential for viral entry are blocked. Via this mechanism, (2) Nabs inhibit the interaction between the TMPRSS2 protease and spike cleavage, essential for the touch of viral and host membranes, or (3) block the membrane fusion and, thus, viral infection. The image was created with https://www.biorender.com/ (accessed on 10 January 2023).

NAbs can be defined as antibodies that bind to the free virus and prevent it from infecting the cells, i.e., they block the binding of the virus to the host cell receptors. The neutralization can happen mainly through three mechanisms: (1) NAbs binding to viral surface proteins and blocking their interaction with the host cell receptor and, hence, the infection; (2) NAbs binding to viral protein epitopes that interact with host cell co-receptors, which are important for viral infection; and (3) NAbs binding to viral epitopes that are not essential for host cell receptor binding but are necessary for the conformational changes needed for membrane fusion [11] (Figure 1).

Although vaccination and natural infection stimulate NAbs production, the vaccines do not avoid viral transmissibility, especially of the BA.2 and BA.5 Omicron lineages and sublineages, such as BQ.1.1 and XBB.1. In vitro studies have indicated a reduced neutralization effect by antibodies from serum obtained from people infected with the ancestral strains or immunized [12,13]. Additionally, other studies showed that neutralization action against the main SARS-CoV-2 variants of concern (VoC) decreased, reducing the protection over time [7,14]. These findings are mainly concerned with SARS-CoV-2 Omicron (B.1.1.529) due to its numerous spike mutations, which is a potential mechanism of evasion from NAbs elicited by COVID-19 vaccines [15]. Recently, in January 2023, a new variant was reported in India. XBB.1.116, named Acturus, is an Omicron subvariant derived from the recombination of BA.2.10.1.1 and BA.2.75.3.1.1.1 sublineages. XBB.1.116 has been reported in at least 33 countries since its discovery. XBB.1.116 was classified as a variant of interest because of its estimated growth advantage compared to other variants and its ability to evade the immune system. However, this variant is associated with mild symptoms with few hospitalizations and deaths [16]. The fast spread of XBB.1.116 associated with immune evasion may contribute to viral replication in vivo and the emergence of new variants.

In contrast to NAbs’ efficacy in serum obtained from infected or vaccinated individuals, a set of specific antibodies have been selected and investigated due their specificity and versatility to neutralize the virus and block its entrance into host cells, acting in both prophylactic and therapeutic applications, respectively [17,18,19]. However, two clinical trials (NCT04427501 and NCT04501978) showed low or no efficacy of monoclonal antibodies (mAbs) for improving clinical outcomes among adults hospitalized with COVID-19 [2,20]. Therefore, measuring the levels of anti-SARS-CoV-2 NAbs in individuals undergoing vaccination and/or who were previously infected remains an indispensable benchmark for assessing acquired protective immunity in the global population. Here, we reviewed the main neutralization assays applied to SARS-CoV-2 NAbs detection and research.

## 2. Methods of Neutralizing Antibody Measurement

### 2.1. Plaque Reduction Neutralization Test—The Gold Standard Assay

The plaque reduction neutralization test (PRNT), developed by Dulbecco and colleagues in 1956, is a functional assay that measures the neutralizing capacity of serum antibodies able to block the virus’ entry and its replication [21,22]. Initially, the PRNT method was used as an assay to follow the evolution of dengue viruses through the measurement of specific NAbs against this disease. Routinely, the researchers assessed the presence of a dengue virus infection by determining NAbs profiles at the individual and population levels [23]. Over time, PRNT was also applied for linking the immune status to clinical outcomes, such as asymptomatic versus symptomatic dengue virus infection, using NAbs as a marker of the immunogenicity after dengue vaccination [24]. Currently, the PRNT assay is considered the gold standard for assessing NAbs titers in cases where the humoral immune response correlates with protection, which is the case for COVID-19.

**Figure 2:**
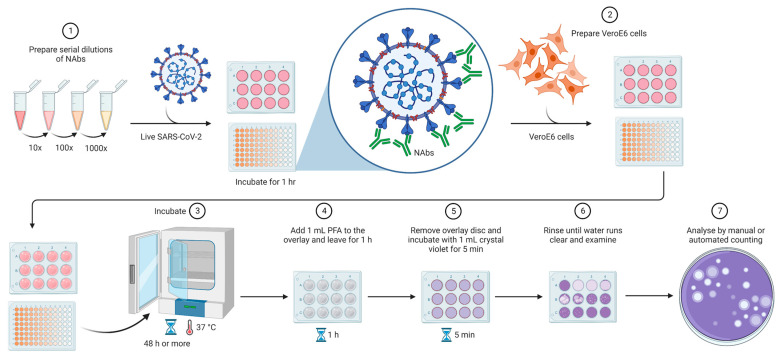
Plaque reduction neutralization test. Samples are serially diluted and incubated with live SARS-CoV-2 for the viral neutralization. VeroE6 cells are added to the system to be infected by non-neutralized viruses. The plates are incubated for at least 48 h, followed by fixation, staining, and counting. The assay can be performed in 24-well plates or smaller plates depending on the throughput to be reached. The analysis can be performed by manual or automated counting. The image was created https://www.biorender.com/ (accessed on 10 January 2023).

In the conventional PRNT, the SARS-CoV-2 measurement is performed by quantifying plaque-forming units (PFU) in specimens containing the antibodies, such as plasma, serum, or blood. In brief, the cells are cultivated in semi-solid media that restrict the spread of the virus to other areas of the plate. When the virus is not neutralized, it can initiate the infection in a localized area, known as plaque, which can be visualized and counted. After the counting of plaques, it is possible to determine the percentage reduction in total virus infection [11,25]. Despite being easy to perform, large foci are difficult to enumerate on an automated immuno spot reader and require additional manual counting and quality control analysis, becoming a low-throughput and disadvantageous approach for large-scale sample screening (Figure 2).

For automated SARS-CoV-2 detection, more sophisticated PRNT protocols have been developed. These processes require susceptible ACE2-expressing cells, NAbs from serum or commercial NAbs, and live viruses. The first step performed is the serial dilution of the test serum or commercial NAbs in an appropriate medium, followed by the addition of the live virus for neutralization. The plates are incubated for a while, and, in case of test serum, if it contains NAbs, the antibodies bind to the virus to form antibody–virus complexes. After that, a volume of the antibody–virus mixture is transferred to the plates containing a monolayer of the ACE2-expressing cells to measure the non-neutralizing virus titer number (Figure 2). To calculate the PRNT_50_, which is characterized as the percentage of viral infection reduction, the percentage reduction in the test serum is compared to that in the non-neutralizing control (only virus, no antibodies) [26,27].

Several clinical studies have shown the accuracy of the PRNT assay for measuring NAbs against SARS-CoV-2 in serum samples from COVID-19-positive or vaccinated individuals, including clinical trials reporting the results for COVID-19 vaccines used nowadays [28,29,30,31,32,33,34,35]. In general, these studies demonstrate a good sensitivity and specificity with no cross-reactivity of the PRNT, guaranteeing accurate results to predict the diagnosis or covering the follow-up of the vaccination against COVID-19. Despite the conventional PRNT assay often being used as the reference method for the evaluation of NAbs against SARS-CoV-2 and other viruses, this assay is time-consuming, laborious, and requires biosafety level 3 (BSL-3) facilities to address the risk of the live viruses [22]. As an alternative, the pseudovirus neutralization test with different spike proteins (Wuhan-Hu-1 reference strain, Beta, Delta, or Omicron and their subvariants) presents fewer requirements, is less expensive, and only requires biosafety level 2 (BSL-2) facilities to conduct the assay, as discussed below [36].

### 2.2. Pseudovirus Neutralization Test 

The pseudovirus neutralization test (pVNT) is a variation of the PRNT test, having the same principle, using pseudotyped viruses instead of live viruses. The pseudotyped viruses are safer non-replicative viral particles, which have a spike as the entry protein, as is found in live SARS-CoV-2. Since the viral particle is non-replicative, it performs only one round of infection. The assay is dependent on ACE2-expressing cells, usually the HEK-293T (human epithelial kidney) cell line, reinforcing the fact that the entry of these particles into cells is dependent on spike/ACE2 interaction [37]. The pseudotyped particles are lentiviruses produced by well-established protocols after the transfection of HEK-293T with backbone and helper plasmids. It is known that the deletion of 18 to 20 C-terminal amino acids improves the pseudovirus titer, and this mutation does not impair viral entry into ACE2-overexpressing HEK cells [38].

**Figure 3:**
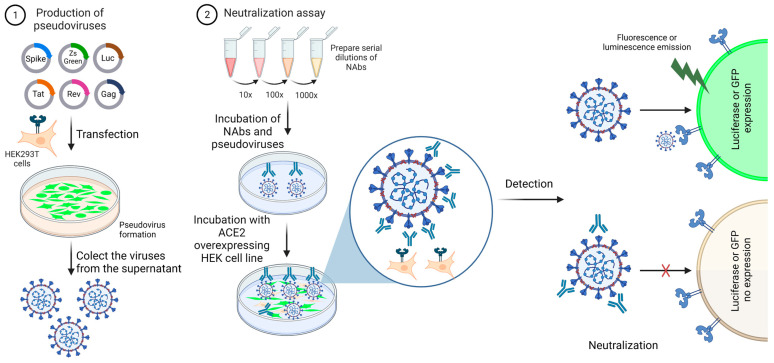
Neutralization assay using pseudoviruses. (1) Lentiviral particles expressing the spike glycoprotein from SARS-CoV-2 and a reporter gene are produced by the HEK-293T cell line. The viral particles are collected from the culture supernatant, filtered, and used for viral titration. (2) Titrated pseudoviruses are used in neutralization assays. The viruses are incubated with serially diluted serum to allow antibody–spike interaction. HEK293T cells overexpressing ACE2 must be used in this assay. The cells are plated and mixed with antibody/viral suspension. Because the pseudoviruses express a reporter gene, cell transduction will generate a transgenic fluorescent cell or a cell able to metabolize the luciferin, for example. This image represents one well from a plate. This assay can be performed at low or high throughput. The image was created with https://www.biorender.com/ (accessed on 10 January 2023).

Except for the virulent viral components and the replicative capacity, the recombinant viral particles contain envelope proteins derived from different viruses, a spike entry protein, and commonly are engineered to carry reporter genes encoding luciferase or green fluorescent protein (GFP) to facilitate the reading out (Figure 3) [37]. The pVNT offers advantages over live-virus-based methods, such as versatility to use different spike proteins related to VoC, safety and sensitivity, and is shown to be in close agreement with PRNT [38,39,40]. In contrast, pVNT has the limitation of mimicking only the viral entry process. To measure the effect on the replication, live viruses are still needed. An additional limitation is that pseudoviral particles are impaired by antiretroviral therapy, including reverse-transcriptase and integrase inhibitors. It causes false-positive results in sera from COVID-19 patients during the treatment with antiretroviral drugs [41,42].

Several studies have applied the pseudovirus neutralization protocol to evaluate the presence of neutralizing antibodies against VoC and measure the potency of plasma from vaccinated volunteers [38,43,44]. A robust serological test using recombinant SARS-CoV-2 pseudovirus for high-throughput assays was developed by Zou and colleagues (2022), and compared to the PRNT reference assay. The pseudovirus produced was composed using a stable mNeonGreen (mNG, a green fluorescence protein), where the mNG gene was engineered at the ORF7 of the viral genome. As a result, both pVNT and PRNT data were obtained using the mNG-positive cells scanned using the CellInsight CX5 high-content screening platform, demonstrating a high correlation (R^2^ = 0.903, *p* < 0.0001) and equal sensibility and specificity. Thus, due to its feasibility, this type of NAbs measurement method using a pseudovirus might be easily adopted [45]. Our research team also developed a high-content imaging-based assay to determine the serum potency of vaccinated people, using the CellInsight CX7 LZR system. The data were validated with a cohort of vaccinated people before vaccination and after one and two doses of CoronaVac, Comirnaty or Covishield. The results demonstrated were correlated with ELISA-based method to detect NAbs (unpublished data).

Most recently, researchers from BioNTech and Pfizer evaluated the sera of 51 participants who received two or three doses of the mRNA-based COVID-19 vaccine BNT162b2 (Pfizer-BioNTech) against Wuhan (*Wuhan*-*Hu*-1 reference strain), Beta (B.1.351), Delta (B.1.617.2), and Omicron pseudoviruses. The results showed high titers of Wuhan reference strain NAbs after two doses of BNT162b2 vaccine. In contrast, with only two doses of the vaccine, low titers were found against the Omicron variant [46]. This work indicated the need for three doses to effectively protect against Omicron-related COVID-19, and the effectiveness of the pVNT assay. This method was also used to demonstrate that the vaccine ChAdOx1 induced multifunctional antibodies after a booster dose [47]. Several other clinical trials have reported the use of pVNT to determine the serum potency via the quantification of viral neutralization [32,48,49,50,51,52,53,54,55,56]. The pVNT can be used as a substitute for the PRNT method in laboratories without BSL3, maintaining the safety and coupling the method with high-content and high-throughput approaches.

### 2.3. Foci Reduction Neutralization Test

**Figure 4:**
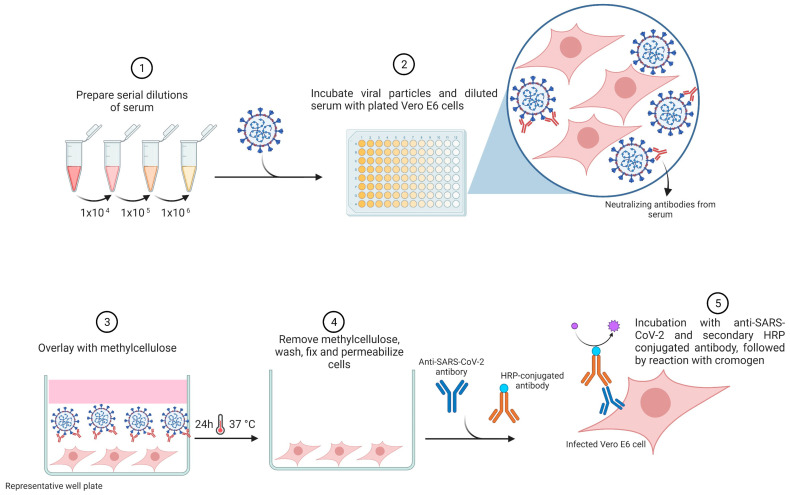
Foci reduction neutralization test (FRNT). Samples are serially diluted and incubated with live SARS-CoV-2 for the viral neutralization for 1 h, followed by incubation with Vero E6 cells for an additional 1 h at 37 °C and 5% CO_2_. Cell culture is overlaid with methylcellulose for 24 h. After removal of the overlay, cells are fixed and permeabilized. Anti-SARS-CoV-2 primary antibody is used to detect infected cells, followed by incubation with secondary HRP-conjugated antibodies. Results are evaluated by an Immunospot analyzer to quantify the spots corresponding to the infected cells. This image represents one well from a plate. This assay can be performed at low or high throughput. The image was created with https://www.biorender.com/ (accessed on 30 June 2023).

The foci reduction neutralization test (FRNT), also called the microneutralization assay (MNA), is an alternative method with a similar principle to the PRNT assay, and it is widely used for NAbs measurement against several viruses [57]. In this assay, SARS-CoV-2-infected cells are determined through an immunostaining procedure using a monoclonal antibody conjugated to horseradish peroxidase (HRP) directed against the spike protein [4]. The results are presented by quantifying the number of foci of SARS-CoV-2-infected cells with a microanalyzer (Figure 4).

Although PRNT is widely used, the FRNT assay has emerged as a powerful option for measuring NAbs against the SARS-CoV-2 virus. In this regard, a recent study reported by Suntronwong and colleagues (2022) evaluated the relationship between NAbs titers against BA.1 and BA.2 Omicron variants by FRNT in a total of 310 serum samples from individuals after booster vaccination. As a result, the NAbs titers obtained from FRNT showed a moderate correlation with the surrogate virus neutralization test (sVNT), which is another assay used for NAbs detection [58].

In accordance with the Food and Drug Administration (FDA), COVID-19 convalescent plasma can be used in the research field to discover new drugs or NAbs against SARS-CoV-2 [59]. In view of this, Annen and colleagues (2021) collected 87 convalescent plasmas, divided into two to four donations from 36 COVID-19-recovered donors, and performed the measurement of SARS-CoV-2 NAbs through FRNT. The data obtained suggested that most donors (67%) had sustained or increased NAbs titers over the short period of approximately 3 weeks to 2 months after a SARS-CoV-2-positive PCR result. On the other hand, a smaller percentage (33%) showed a decrease in NAbs titers over time. Furthermore, the findings showed that 83% of donors had a FRNT_50_ titer of at least 80, and 61% had a titer of at least 160, which are considered acceptable by the FDA as a source of NAbs [59]. This method has been used in clinical trials to access NAbs in vaccinated people [32,48,55]. Although the FRNT method can be performed easily to measure the NAbs and is high-throughput compatible, it still requires live viruses, thus presenting safety concerns [60].

### 2.4. Surrogate Virus Neutralization Test—An ELISA-Based Approach

**Figure 5:**
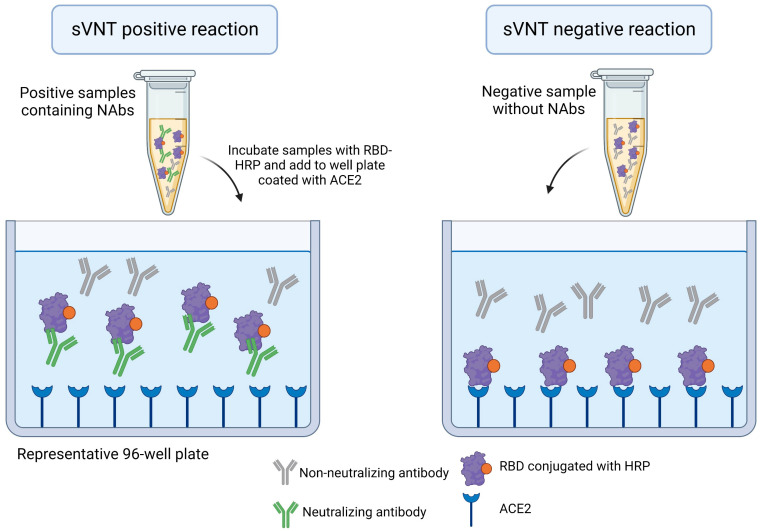
Surrogate virus neutralization test (sVNT). This method is an ELISA-based approach. Samples are incubated with a recombinant receptor-binding domain (RBD) of spike conjugates to horseradish peroxidase (HRP). Neutralizing antibodies, if present in the sample, will bind to RBD-HRP. This interaction abolishes the binding of RBD-HRP to angiotensin-converting enzyme 2 (ACE2), in a 96-well plate. The incubation with HRP substrate will generate a signal, which is inversely proportional to the NAbs titer. This image represents one well from a plate. This assay can be performed at low or high throughput. The image was created with https://www.biorender.com/ (accessed on 30 June 2023).

The surrogate virus neutralization test (sVNT) replicates the virus–host interaction by incubating a sample (serum, plasma, or blood) with a purified viral spike receptor binding domain (RBD) conjugated to HRP, and immobilized human ACE2 receptors in an enzyme linked immunosorbent Assay (ELISA) plate. In cases where NAbs are present in the sample, the HRP-conjugated RBD will not bind to human ACE2, and there will be no or a lower-intensity signal, and vice-versa (Figure 5) [11]. This method is safe, since it used recombinant protein, and has a reduced cost compared to cell-based and live-virus methods.

Following this approach, a competitive immunoassay with electrochemiluminescence was developed by Sancilio and colleagues, and its performance was determined. This assay was easy to perform and used a dried blood spot as the sample, characterizing it as a minimally invasive method. The presented outcomes showed that the use of a dried blood spot generated results, which were closely related to serum use. The percentage of neutralization found in PCR-confirmed convalescent samples was 46.9%, compared to 0.1% found in negative samples. The sVNT presented 74.5% sensitivity. However, if the tested samples were from a PCR-positive person who had mild or asymptomatic COVID-19, the sensitivity dropped to 18.8% [61].

In a study performed by Tan and colleagues (2020), a sVNT version that detects NAbs without the need for any live virus or cells was developed. Using purified RBD from the spike protein and the host cell ACE2 receptor, this assay might mimic the virus–host interaction in an ELISA microplate, being compatible with high-throughput. If NAbs are detected in the samples tested, the RBD–ACE2 interaction can be neutralized. Thus, to validate this method, samples provided from two cohorts of COVID-19 positive patients in two different countries were evaluated. As a result, 99.93% specificity and 95–100% sensitivity was observed, indicating that this sVNT assay is promising [62]. Other studies have reposted similar results by applying the sVNT, including a clinical trial evaluating the immunogenicity of an extended dosing interval of BNT162b2 against SARS-CoV-2 Omicron among healthy school-aged children [49,63,64,65]. It is noteworthy that NAbs binding to spike S1 subunits outside the RBD motif are no longer evaluated using the method reported above [6,66]. Therefore, this approach may lose information about these S1-specific antibodies, which can also neutralize SARS-CoV-2.

## 3. Innovative Strategies for the Detection and Quantification of SARS-CoV-2 Neutralizing Antibodies

The main articles reported in this review are summarized in Table 1. However, considering that neutralization assays are powerful tools used for COVID-19 diagnosis and the monitoring of vaccine-mediated protection, innovative strategies have emerged aiming to detect and measure accurately the neutralizing potential of antibodies against SARS-CoV-2. Currently, besides the methods mentioned above, different strategies, such as the lateral flow immunoassay (LFIA), ELISA, and surface plasmon resonance have been used to detect SARS-CoV-2 NAbs [67,68,69,70,71,72].

Despite the great advances made with conventional methods, the gold-standard method requires BSL-3 due to the high risk of exposure to and transmission of the pathogen. The pVNT requires BSL-2 and can overcome this limitation [9]. Under the conditions of live virus use, expensive infrastructure and the recruitment of highly qualified human resources to work at a BSL-3 laboratory are required. Some methods more recently developed, such as sVNT and a rapid LFIA, present with good performance; however, they cannot detect all NAbs in the patient’s sample (in case the full-length spike is not used), and present variable neutralization titer values [9]. Additionally, although PRNT and ELISA generate correlated results with each other, PRNT is still the gold standard method, even it has a low throughput and it is not practical for large-scale serodiagnosis and vaccine monitoring, generating a gap for COVID-19 surveillance and vaccine development [73]. Taken together, all these drawbacks reinforce the necessity to optimize or develop alternative tools, especially because highly accurate serological tests are essential for assessing the prevalence of neutralizing SARS-CoV-2 antibodies and the level of humoral immunity in the population.

Among the novel approaches, nanotechnology presents a variety of applications to improve SARS-CoV-2 neutralization in vivo. It includes the ACE2-based nano decoys as a strategy [75]. This nano-based method involves the preparation of ACE2 receptor-modified decoy nanoparticles (NPs) to neutralize different SARS-CoV-2 variants. Like host cells containing ACE2 in their membrane, these nano decoys are engineered with ACE2 receptors on their surface, allowing them to bind covalently to the viruses in vivo improving the neutralization. Nano decoys could be adapted to detect NAbs, following the use of spike-based nanostructures. It has been done in LFIA platforms, for example. Additionally, since the LFIA platforms is a cell-free approach, it can be desirable for a quick assay and high-throughput screenings. This nanotechnological approach was used by Bian and colleagues (2022) to establish an ultrabright nanoparticle-based LFIA for the one-step rapid semi-quantitative detection of anti-SARS-CoV-2 NAbs. An aggregation-induced emission (AIE) luminogen, AIE_490_, conjugated to the polystyrene nanoparticles (AIE_490_NP), was used as a marker of LFIA. The AIE_490_NP was functionalized with ACE2 (ACE2-AIE_490_NP) as a fluorescence marker located in the conjugation pad of the LFIA, while the spike protein was coated to a nitrocellulose membrane, as a teste line. Control line was represented by an anti-human IgG antibody, which recognizes the AIE_490_NP conjugated to human IgG from the conjugation pad. When the sample is negative for NAbs, the test line exhibits a bright fluorescence signal because of the strong ACE2-spike interaction; while, if the sample is positive for NAbs, the test line presents a dim fluorescence signal since the NAbs-spike interaction inhibits the binding between ACE2-AIE_490_NP and spike. To verify its performance, 70 negative and 63 positive samples of human serum were evaluated, and the detection of fluorescence signal intensity was performed and quantified to determine the threshold detection of the AIE490NP-based LFIA. As a result, the detection method threshold was calculated and capable to distinguish between negative and positive samples containing NAbs. The AIE_490_NP-based LFIA presented an acceptable reproducibility. The intra- and inter-assay reproducibility was around 9.37–12.99% and 12.69–15.03% for negative samples, respectively. The intra- and inter-assay reproducibility regarding positive samples were 6.81–7.58% and 7.15–8.44%, respectively. Considering the highest coefficient of variance as 15% the test presented acceptable reproducibility. Therefore, the authors suggested the AIE_490_NP-based LFIA as an alternative method for the rapid detection of NAbs levels in vaccinated sera [68].

Measuring the SARS-CoV-2 NAbs is crucial for the precise monitoring of serological epidemiology and to determine infection control of SARS-CoV-2. ELISA and LFIA assays are widely used serological methods, but they present relatively low sensitivity and a high rate of false positives, especially because they do not distinguish between NAbs and non-NAbs [79]. As an alternative method, Tani and colleagues (2021) developed an antibody detection system based on the chemiluminescence reduction neutralization test (CRNT), using the truncated spike protein-based pseudotyped viruses. CRNT assay presented promising results and revealed advantages over ELISA and LFIA, such as safety and celerity in the assay processing [78].

Another concern for the development of methods to detect NAbs is the non-specific interactions with other antibodies in the serological assays, which might cause erroneous analyses. In this sense, Mravinacova and colleagues (2022) developed a high-throughput method to assess the ability of the anti-spike antibodies to neutralize the spike protein’s interaction with ACE2. In this method serum samples are preincubated with biotinylated recombinant spike, followed by the incubation with ACE2-coupled magnetic beads. Absence of NAbs allow the spike-ACE2 binding. Fluorescently labelled streptavidin is added to enable read out of bead-bound spike. In contrast with other less specific serological methods that measure all antibodies against the spike protein, Mravinacova’s test precisely detect NAbs. This assay is cell-free, using only a spike, which has a trimeric structure stabilized by exchanging the furin cleavage site for two prolines, and an ACE2 receptor to mimic the binding; the assay allows analysis of up to 384 samples at the same time requiring seven hours, including one hour of manual handling, to analyze a 384-well plate. Moreover, the assay can be adapted to the most recent VoC and determined the neutralization capacity of such population against circulating variants. Compared with the microneutralization method, this novel bead-based strategy reproduces comparable data to determine neutralization, but without the need to cultivate live viruses and cells (during the assay execution) [74].

Indeed, the use of cell-free methods to detect NAbs have been identified as a great tool to better track herd immunity, vaccine efficacy and vaccination rates. Dahn and colleagues (2022) reported the split oligonucleotide neighboring inhibition assay (SONIA) strategy, which uses real-time qPCR to measure the capacity of NAbs to block the binding between DNA-barcoded viral spike protein subunit 1 (S1) and the ACE2 receptor in dried blood spots. Despite having shown 91–97% sensitivity and 100% specificity in comparison with the standard methods, as well as the advantages such as no washing, centrifugation or cell culturing, FDA authorization has not been provided for SONIA or any other assay for measuring NAbs in dried blood spots currently. However, this novel method is a promising assay that could be adopted in the future [76].

High-throughput NAbs assays are urgently required for a robust and rapid COVID-19 diagnosis, vaccine monitoring and mAB screening. In this sense, Muruato and colleagues (2020) developed a fluorescence-based neutralization assay that detects SARS-CoV-2 NAbs in COVID-19 patient specimens and yields comparable results to PRNT. To validate this high-throughput assay, the researchers tested the mNeonGreen SARS-CoV-2 virus. Nevertheless, it is necessary to use BSL-3 to perform the test. In this work, a robust NAbs quantifier method using the CellInsight CX5 high-content screening platform was described. According to this platform’s instructions, with predefined threshold parameters obtained using non-infected and pseudovirus-infected cells, in a few minutes, a 96-well microplate can be analyzed and scanned through a rapid fluorescence-based high-throughput assay [73]. Despite the limitations of non-specific signals always existing in high-content imaging due to auto-fluorescence from factors, including environmental dust, plastics, and dead cells, the right set up of parameters and the optimization of precise adjustments make a rapid and very robust analysis possible [45].

To conduct a large-scale evaluation of the frequency and potency of NAbs titers in SARS-CoV-2-infected or vaccinated populations, it is necessary to design a fast, affordable, sensitive, and quantitative SARS-CoV-2 NAbs detection assay. In this sense, Huang and colleagues (2021) developed a rapid (<30 min), sensitive, cell-free, off-the-shelf and accurate assay for RBD NAbs detection; this alternative method is a great option when the PRNT assay is not adequate, as that requires at least two days to carry out [41]. In general, this approach takes advantage of NanoLuc^®^ Binary technology, which presents a proximity-based luciferase system, and is precise and robust to detect NAbs. NanoLuc^®^ Binary consists of two separated proteins, Large BiT (LgBiT, 18 kDa) and small complementary BiT (SmBiT, 11 aa), which generating an active luciferase when interact with appropriate geometry. SmBiT was expressed as a fused protein to the N-terminus of spike-S1 domain, while LgBiT was expressed fused to ACE-2. In the absence of NAbs, spike-ACE2 interaction allow the contact between Large BiT and SmBiT, generating a luciferase able to emit light in the presence of substrate. When NAbs are present in the sample, spike-ACE2 binding is impaired, and less bioluminescence is produced. The method is compatible to high throughput and can be applied in the screening of NAbs against variant of concerns [41].

An approach based on cyclic voltammetry is other innovative strategy to detect NAbs against SARS-CoV-2. The system emits the signal of an impedimetric biosensor, constructed based on the immobilization of the SARS-CoV-2 spike protein on zinc oxide nanorod (ZnONR) electrodes. When working at the physiological pH equal to 7.4, spike glycoprotein presents a net negative surface charge (isoelectric point of approximately 5), while the ZnONR, which has a high isoelectric point of approximately 9.5, presents a net positive surface charge density. It allows the immobilization of spike on the surface of the ZnONR matrix. The binding of NAbs to the immobilized spike increases the signal detected by the sensor, which is attributed to the bulky-sized antibodies covering the sensor surface. The signal detected is a consequence of the reduced electron transfer efficiency by the ferro/ferricyanide redox couple. This system was developed to detect convalescent antibodies against the virus or NAbs and can be adapted to screen antibodies against VoC, since the immobilized spike had been changed. An ELISA to detect IgG against SARS-CoV-2 was used to measure the sensitivity, while the measurement of IgG, by ELISA, against seasonal coronavirus (HCoVs) was used to determine the specificity of the assay. The biosensor technology presented 100% of specificity and 88.7% of sensitivity. These electrodes represent a point of care assay do determine the levels of NAbs against SARS-CoV-2, being an excellent tool for monitoring the seroprevalence and surveillance [77].

## 4. Perspectives and Conclusions

Overall, all conventional and novel methods present advantages and limitations, as shown in Table 2, for accurately assessing the neutralization efficacy of NAbs. Almost all novel methods are nano-based and cell-free and were developed to be more practical and faster than conventional methods. However, none of them can measure the replicative effect of live viruses or NAbs targeting RBD-outside sites. Therefore, there is still a need to develop and invest in new approaches for NAbs measurement. As a perspective, tools for in silico assays have emerged and been used to develop highly potent NAbs that broadly target all currently circulating SARS-CoV-2 variants [80]. The aim of using these bioinformatic platforms is to identify vulnerable target sites on coronaviruses for the development of potential NAbs and to assess vaccine efficacy. Additionally, with the use of computational datasets of patients or cohorts, it is possible to analyze specific NAbs epitopes and accurately estimate the precision of their efficacy in inducing immunity [81].

In conclusion, many approaches have been used to improve the quantification of NAbs; however, there is no perfect assay, and all of them present limitations regarding safety, throughput, or the antibody targeting site. An alternative to overcome this scenario is to apply more than one validated system, such as a pVNT, followed by a PRNT, only if a BSL-3 structure is available. On the other hand, two live-virus-independent methodologies can be used, considering the presence of a full-length spike. These strategies aim to make the neutralization assays as close as possible to the real biological event to determine the amount of NAbs with high accuracy.

## Figures and Tables

**Table 1:** Main articles reported and the methods used to determine NAbs.

Main Articles Reported	References
These works applied the PRNT ^1^ as a reference to compare the performance of other methods, such as the microneutralization assay, surrogate virus neutralization test and pseudotyped virus neutralization assay. The authors showed the main advantages and limitations of each method	[27,29]
This work applied PRNT to measure the beneficial effect of convalescent plasma treatment of critically ill patients. They used PRNT to determine viral neutralization	[28]
Safety and immunogenicity studies of COVID-19 vaccines which were subjected to the PRNT assay	[30,31,32,33,34,35]
Works that applied pVNT ^2^ to measure NAbs ^3^, including those in which the immunogenicity of COVID-19 vaccines was evaluated	[32,38,43,44,45,46,47,48,49,50,51,52,53,54,55,56,73]
Works that applied FRNT ^4^, including clinical trials	[4,32,48,53,58,59]
sVNT ^5^ reported, including a randomized clinical trial	[33,49,62]
SARS-CoV-2 neutralization assays based on LFIA ^6^	[72]
SARS-CoV-2 neutralization assays based on SPR ^7^	[69,70]
SARS-CoV-2 neutralization assays based on MIA ^8^	[71,74]
Nanotechnology-based neutralization assays	[41,68,75,76,77]
SARS-CoV-2 neutralization assays based CRNT ^9^	[78]

^1^ Plaque reduction neutralization assay; ^2^ pseudovirus neutralization test; ^3^ neutralizing antibodies; ^4^ foci reduction neutralization test; ^5^ surrogate virus neutralization tests; ^6^ lateral flow immunoassays; ^7^ surface plasmon resonance; ^8^ microsphere immunoassay; ^9^ chemiluminescence reduction neutralization test.

**Table 2:** Principle, advantages, and limitations of NAbs detection assays.

Assay	Principle	Advantages	Limitations
Plaque reduction neutralization test (PRNT)	Infection of Vero cells with live viruses. The measurement of NAbs’ potency is determined through the quantification of the plaque-forming units (PFU) in specimens containing the antibodies, such as plasma, serum, or blood.	Since PRNT uses live viruses, the replication capacity of the virus is considered.	Safety concerns regarding the use of live viruses. Laboratorial infrastructure (BSL-3) to use live viruses. Highly qualified human resources to work in the BSL-3 laboratory. PRNT is not compatible with high throughput, time-consuming, laborious.
Pseudovirus neutralization test (pVNT)	Production of lentivirus expressing spike in the surface and transduction of HEK cells overexpressing ACE2. HEK cells are transduced to express GFP or luciferase. The redout is based on the reduction of the percentage of GFP positive cells of the decrease in the luciferase bioluminescence.	It requires BSL-2. Compatible with high throughput. Can be quickly adapted to produce lentiviral particles expressing spike from VoC.	Since pVNT uses non-replicative viral particles, viral replication is not considered in this assay. Evaluates only spike-ACE-2 interaction. Antiretroviral treatment may produce false positive results regarding pseudoviral neutralization. pVNT requires ACE2-expressing cells to measure anti-SARS-CoV-2 NAbs.
Foci reduction neutralization test (FRNT) or microneutralization assay (MNA)	FRNT has a similar principle to the PRNT assay. However, the readout is based on a monoclonal antibody conjugated to horseradish peroxidase (HRP) directed against the spike protein. This method uses live viruses. The PFU is quantified by a microanalyzer.	Since PRNT uses live viruses, the replication capacity of the virus is considered. Compatible with high throughput.	Safety concerns regarding the use of live viruses. Laboratorial infrastructure (BSL-3) to use live viruses. Highly qualified human resources to work in the BSL-3 laboratory.
Surrogate virus neutralization test (sVNT)	The method is based on an enzyme linked immunosorbent Assay (ELISA) and measures the RBD-ACE2 interaction. The RBD conjugates with HRP interacts with ACE2 in an ELISA plate in the absence of NAbs	sVNT is easy to execute. sVNT presents less safety concern compared to PRNT and pVNT, since it uses recombinant protein. It has a reduced cost compared to cell-based and’ live-virus methods. Can be quickly adapted to produce lentiviral particles ex-pressing spike from VoC. Compatible with high throughput.	The assay evaluates RBD-ACE2 interaction. sVNT may lose NAbs binding outside the RBD
Lateral flow immunoassay (LFIA)	The assay uses RBD or full-length spike conjugated to a colloidal gold, colorful microsphere or fluorescent beads. NAbs from the sample interact with RBD in the conjugation pad. NAbs-RBD binding will avoid RBD binding to ACE2 coated in the test line. In this case the test line will be less colorful, or less fluorescent depending on the NAbs titer.	Easy to execute test. Usually, it takes 10 to 15 min to see the result.	The use of conjugated RBD may miss NAbs targeting spike domains outside the RBD
		It can be performed in laboratories with high or low infrastructure. It can be used in places without laboratorial infrastructure. Can be adapted for point of care tests. Can be adapted for different readouts, such as colloidal gold, microspheres and fluorescence. LFIA is a cell-free platform and a cheap method. The assay can be qualitative or quantitative. It can be adapted to high throughput screenings.	It requires high binding affinity between the target protein and its receptor (RBD and ACE2). Most LFIA are qualitative and to become quantitative it must be associated with an equipment or system (like mobile phones) to measure the intensity of the test and control lines.

Biosafety level (BSL); green fluorescent protein (GFP); receptor binding domains (RBD); Angiotensin-converting enzyme 2 (ACE2); horseradish peroxidase (HRP); neutralizing antibodies (NAbs).

## Data Availability

Not applicable.

## References

[1] WHO Coronavirus (COVID-19) Dashboard|WHO Coronavirus (COVID-19) Dashboard with Vaccination Data. https://covid19.who.int/.

[2] Chen P., Nirula A., Heller B., Gottlieb R.L., Boscia J., Morris J., Huhn G., Cardona J., Mocherla B., Stosor V. (2021). SARS-CoV-2 Neutralizing Antibody LY-CoV555 in Outpatients with COVID-19. N. Engl. J. Med..

[3] Shang J., Wan Y., Luo C., Ye G., Geng Q., Auerbach A., Li F. (2020). Cell Entry Mechanisms of SARS-CoV-2. Proc. Natl. Acad. Sci. USA.

[4] Vanderheiden A., Edara V.V., Floyd K., Kauffman R.C., Mantus G., Anderson E., Rouphael N., Edupuganti S., Shi P.Y., Menachery V.D. (2020). Development of a Rapid Focus Reduction Neutralization Test Assay for Measuring SARS-CoV-2 Neutralizing Antibodies. Curr. Protoc. Immunol..

[5] Machado B.A.S., Hodel K.V.S., Fonseca L.M.D.S., Pires V.C., Mascarenhas L.A.B., da Silva Andrade L.P.C., Moret M.A., Badaró R. (2022). The Importance of Vaccination in the Context of the COVID-19 Pandemic: A Brief Update Regarding the Use of Vaccines. Vaccines.

[6] Castro Dopico X., Ols S., Loré K., Karlsson Hedestam G.B. (2022). Immunity to SARS-CoV-2 Induced by Infection or Vaccination. J. Intern. Med..

[7] Khoury D.S., Cromer D., Reynaldi A., Schlub T.E., Wheatley A.K., Juno J.A., Subbarao K., Kent S.J., Triccas J.A., Davenport M.P. (2021). Neutralizing Antibody Levels Are Highly Predictive of Immune Protection from Symptomatic SARS-CoV-2 Infection. Nat. Med..

[8] Counoupas C., Pino P., Stella A.O., Ashley C., Lukeman H., Bhattacharyya N.D., Tada T., Anchisi S., Metayer C., Martinis J. (2022). High-Titer Neutralizing Antibodies against the SARS-CoV-2 Delta Variant Induced by Alhydroxyquim-II-Adjuvanted Trimeric Spike Antigens. Microbiol. Spectr..

[9] Liu K.T., Han Y.J., Wu G.H., Huang K.Y.A., Huang P.N. (2022). Overview of Neutralization Assays and International Standard for Detecting SARS-CoV-2 Neutralizing Antibody. Viruses.

[10] Baum A., Ajithdoss D., Copin R., Zhou A., Lanza K., Negron N., Ni M., Wei Y., Mohammadi K., Musser B. (2020). REGN-COV2 Antibodies Prevent and Treat SARS-CoV-2 Infection in Rhesus Macaques and Hamsters. Science.

[11] Morales-Núñez J.J., Muñoz-Valle J.F., Torres-Hernández P.C., Hernández-Bello J. (2021). Overview of Neutralizing Antibodies and Their Potential in COVID-19. Vaccines.

[12] Planas D., Veyer D., Baidaliuk A., Staropoli I., Guivel-Benhassine F., Rajah M.M., Planchais C., Porrot F., Robillard N., Puech J. (2021). Reduced Sensitivity of SARS-CoV-2 Variant Delta to Antibody Neutralization. Nature.

[13] Cromer D., Steain M., Reynaldi A., Schlub T.E., Wheatley A.K., Juno J.A., Kent S.J., Triccas J.A., Khoury D.S., Davenport M.P. (2022). Neutralising Antibody Titres as Predictors of Protection against SARS-CoV-2 Variants and the Impact of Boosting: A Meta-Analysis. Lancet Microbe.

[14] Wang P., Nair M.S., Liu L., Iketani S., Luo Y., Guo Y., Wang M., Yu J., Zhang B., Kwong P.D. (2021). Antibody Resistance of SARS-CoV-2 Variants B.1.351 and B.1.1.7. Nature.

[15] Pérez-Then E., Lucas C., Monteiro V.S., Miric M., Brache V., Cochon L., Vogels C.B.F., Malik A.A., De la Cruz E., Jorge A. (2022). Neutralizing Antibodies against the SARS-CoV-2 Delta and Omicron Variants Following Heterologous CoronaVac plus BNT162b2 Booster Vaccination. Nat. Med..

[16] Looi M.K. (2023). What Do We Know about the Arcturus XBB.1.16 Subvariant?. BMJ.

[17] Hwang Y.C., Lu R.M., Su S.C., Chiang P.Y., Ko S.H., Ke F.Y., Liang K.H., Hsieh T.Y., Wu H.C. (2022). Monoclonal Antibodies for COVID-19 Therapy and SARS-CoV-2 Detection. J. Biomed. Sci..

[18] Wang C., Li W., Drabek D., Okba N.M.A., van Haperen R., Osterhaus A.D.M.E., van Kuppeveld F.J.M., Haagmans B.L., Grosveld F., Bosch B.J. (2020). A Human Monoclonal Antibody Blocking SARS-CoV-2 Infection. Nat. Commun..

[19] Baral P.K., Yin J., James M.N.G. (2021). Treatment and Prevention Strategies for the COVID 19 Pandemic: A Review of Immunotherapeutic Approaches for Neutralizing SARS-CoV-2. Int. J. Biol. Macromol..

[20] Self W.H., Sandkovsky U., Reilly C.S., Vock D.M., Gottlieb R.L., Mack M., Golden K., Dishner E., Vekstein A., Ko E.R. (2022). Efficacy and Safety of Two Neutralising Monoclonal Antibody Therapies, Sotrovimab and BRII-196 plus BRII-198, for Adults Hospitalised with COVID-19 (TICO): A Randomised Controlled Trial. Lancet Infect. Dis..

[21] Schmidt N.J., Lennette E.H. (1961). Virology on the Bookshelf. Application of Tissue Culture Technics to Diagnostic Virology in the Public Health Laboratory. Am. J. Public. Health Nations Health.

[22] Valcourt E.J., Manguiat K., Robinson A., Lin Y.-C., Abe K.T., Mubareka S., Shigayeva A., Zhong Z., Girardin R.C., DuPuis A. (2021). Evaluating Humoral Immunity against SARS-CoV-2: Validation of a Plaque-Reduction Neutralization Test and a Multilaboratory Comparison of Conventional and Surrogate Neutralization Assays. Microbiol. Spectr..

[23] Russell P.K., Nisalak A., Sukhavachana P., Vivona S. (1967). A Plaque Reduction Test for Dengue Virus Neutralizing Antibodies. J. Immunol..

[24] Thomas S.J., Nisalak A., Anderson K.B., Libraty D.H., Kalayanarooj S., Vaughn D.W., Putnak R., Gibbons R.V., Jarman R., Endy T.P. (2009). Dengue Plaque Reduction Neutralization Test (PRNT) in Primary and Secondary Dengue Virus Infections: How Alterations in Assay Conditions Impact Performance. Am. J. Trop. Med. Hyg..

[25] Mendoza E.J., Manguiat K., Wood H., Drebot M. (2020). Two Detailed Plaque Assay Protocols for the Quantification of Infectious SARS-CoV-2. Curr. Protoc. Microbiol..

[26] Maeda A., Maeda J. (2013). Review of Diagnostic Plaque Reduction Neutralization Tests for Flavivirus Infection. Vet. J..

[27] Bewley K.R., Coombes N.S., Gagnon L., McInroy L., Baker N., Shaik I., St-Jean J.R., St-Amant N., Buttigieg K.R., Humphries H.E. (2021). Quantification of SARS-CoV-2 Neutralizing Antibody by Wild-Type Plaque Reduction Neutralization, Microneutralization and Pseudotyped Virus Neutralization Assays. Nat. Protoc..

[28] Shen C., Wang Z., Zhao F., Yang Y., Li J., Yuan J., Wang F., Li D., Yang M., Xing L. (2020). Treatment of 5 Critically Ill Patients With COVID-19 With Convalescent Plasma. JAMA.

[29] Mahmoud S.A., Ganesan S., Naik S., Bissar S., Al Zamel I., Warren K., Zaher W.A., Khan G. (2021). Serological Assays for Assessing Postvaccination SARS-CoV-2 Antibody Response. Microbiol. Spectr..

[30] Anderson E.J., Rouphael N.G., Widge A.T., Jackson L.A., Roberts P.C., Makhene M., Chappell J.D., Denison M.R., Stevens L.J., Pruijssers A.J. (2020). Safety and Immunogenicity of SARS-CoV-2 MRNA-1273 Vaccine in Older Adults. N. Engl. J. Med..

[31] Mulligan M.J., Lyke K.E., Kitchin N., Absalon J., Gurtman A., Lockhart S., Neuzil K., Raabe V., Bailey R., Swanson K.A. (2020). Phase I/II Study of COVID-19 RNA Vaccine BNT162b1 in Adults. Nature.

[32] Folegatti P.M., Ewer K.J., Aley P.K., Angus B., Becker S., Belij-Rammerstorfer S., Bellamy D., Bibi S., Bittaye M., Clutterbuck E.A. (2020). Safety and Immunogenicity of the ChAdOx1 NCoV-19 Vaccine against SARS-CoV-2: A Preliminary Report of a Phase 1/2, Single-Blind, Randomised Controlled Trial. Lancet.

[33] Leung N.H.L., Cheng S.M.S., Martín-Sánchez M., Au N.Y.M., Ng Y.Y., Luk L.L.H., Chan K.C.K., Li J.K.C., Leung Y.W.Y., Tsang L.C.H. (2023). Immunogenicity of a Third Dose of BNT162b2 to Ancestral Severe Acute Respiratory Syndrome Coronavirus 2 and the Omicron Variant in Adults Who Received 2 Doses of Inactivated Vaccine. Clin. Infect. Dis..

[34] Sanders J.-S.F., Messchendorp A.L., de Vries R.D., Baan C.C., van Baarle D., van Binnendijk R., Diavatopoulos D.A., Geers D., Schmitz K.S., GeurtsvanKessel C.H. (2023). Antibody and T-Cell Responses 6 Months after Coronavirus Disease 2019 Messenger RNA-1273 Vaccination in Patients with Chronic Kidney Disease, on Dialysis, or Living with a Kidney Transplant. Clin. Infect. Dis..

[35] Valim V., Martins-Filho O.A., Gouvea M.d.P.G., Camacho L.A.B., Villela D.A.M., de Lima S.M.B., Azevedo A.S., Neto L.F.P., Domingues C.M.A.S., de Medeiros Junior N.F. (2022). Effectiveness, Safety, and Immunogenicity of Half Dose ChAdOx1 NCoV-19 COVID-19 Vaccine: Viana Project. Front. Immunol..

[36] Banga Ndzouboukou J.-L., Zhang Y.-D., Fan X.-L. (2021). Recent Developments in SARS-CoV-2 Neutralizing Antibody Detection Methods. Curr. Med. Sci..

[37] Crawford K.H.D., Eguia R., Dingens A.S., Loes A.N., Bloom J.D., Crawford K. (2021). Pseudotyping Lentiviral Particles with SARS-CoV-2 Spike Protein for Neutralization Assays. Protocols.io.

[38] Garcia-Beltran W.F., Lam E.C., St Denis K., Nitido A.D., Garcia Z.H., Hauser B.M., Feldman J., Pavlovic M.N., Gregory D.J., Poznansky M.C. (2021). Multiple SARS-CoV-2 Variants Escape Neutralization by Vaccine-Induced Humoral Immunity. Cell.

[39] Nie J., Li Q., Wu J., Zhao C., Hao H., Liu H., Zhang L., Nie L., Qin H., Wang M. (2020). Establishment and Validation of a Pseudovirus Neutralization Assay for SARS-CoV-2. Emerg. Microbes Infect..

[40] Case J.B., Rothlauf P.W., Chen R.E., Liu Z., Zhao H., Kim A.S., Bloyet L.M., Zeng Q., Tahan S., Droit L. (2020). Neutralizing Antibody and Soluble ACE2 Inhibition of a Replication-Competent VSV-SARS-CoV-2 and a Clinical Isolate of SARS-CoV-2. Cell Host Microbe.

[41] Huang D., Tran J.T., Peng L., Yang L., Suhandynata R.T., Hoffman M.A., Zhao F., Song G., He W., Limbo O. (2021). A Rapid Assay for SARS-CoV-2 Neutralizing Antibodies That Is Insensitive to Antiretroviral Drugs. J. Immunol..

[42] Powell S.K., Artlip M., Kaloss M., Brazinski S., Lyons R., McGarrity G.J., Otto E. (1999). Efficacy of Antiretroviral Agents against Murine Replication-Competent Retrovirus Infection in Human Cells. J. Virol..

[43] Garcia-Beltran W.F., St Denis K.J., Hoelzemer A., Lam E.C., Nitido A.D., Sheehan M.L., Berrios C., Ofoman O., Chang C.C., Hauser B.M. (2022). MRNA-Based COVID-19 Vaccine Boosters Induce Neutralizing Immunity against SARS-CoV-2 Omicron Variant. Cell.

[44] Garcia-Beltran W.F., Lam E.C., Astudillo M.G., Yang D., Miller T.E., Feldman J., Hauser B.M., Caradonna T.M., Clayton K.L., Nitido A.D. (2021). COVID-19-Neutralizing Antibodies Predict Disease Severity and Survival. Cell.

[45] Zou J., Xia H., Shi P.Y., Xie X., Ren P. (2022). A Single-Round Infection Fluorescent SARS-CoV-2 Neutralization Test for COVID-19 Serological Testing at a Biosafety Level-2 Laboratory. Viruses.

[46] Muik A., Lui B.G., Wallisch A.K., Bacher M., Mühl J., Reinholz J., Ozhelvaci O., Beckmann N., Garcia R.d.l.C.G., Poran A. (2022). Neutralization of SARS-CoV-2 Omicron by BNT162b2 MRNA Vaccine-Elicited Human Sera. Science.

[47] Barrett J.R., Belij-Rammerstorfer S., Dold C., Ewer K.J., Folegatti P.M., Gilbride C., Halkerston R., Hill J., Jenkin D., Stockdale L. (2021). Phase 1/2 Trial of SARS-CoV-2 Vaccine ChAdOx1 NCoV-19 with a Booster Dose Induces Multifunctional Antibody Responses. Nat. Med..

[48] Niyomnaitham S., Jongkaewwattana A., Meesing A., Pinpathomrat N., Nanthapisal S., Hirankarn N., Siwamogsatham S., Kirdlarp S., Chaiwarith R., Lawpoolsri S. (2023). Immunogenicity of a Fractional or Full Third Dose of AZD1222 Vaccine or BNT162b2 Messenger RNA Vaccine after Two Doses of CoronaVac Vaccines against the Delta and Omicron Variants. Int. J. Infect. Dis..

[49] Chantasrisawad N., Techasaensiri C., Kosalaraksa P., Phongsamart W., Tangsathapornpong A., Jaru-Ampornpan P., Sophonphan J., Suntarattiwong P., Puthanakit T. (2023). The Immunogenicity of an Extended Dosing Interval of BNT162b2 against SARS-CoV-2 Omicron Variant among Healthy School-Aged Children, a Randomized Controlled Trial. Int. J. Infect. Dis..

[50] Ali K., Berman G., Zhou H., Deng W., Faughnan V., Coronado-Voges M., Ding B., Dooley J., Girard B., Hillebrand W. (2021). Evaluation of MRNA-1273 SARS-CoV-2 Vaccine in Adolescents. N. Engl. J. Med..

[51] Borobia A.M., Carcas A.J., Pérez-Olmeda M., Castaño L., Bertran M.J., García-Pérez J., Campins M., Portolés A., González-Pérez M., García Morales M.T. (2021). Immunogenicity and Reactogenicity of BNT162b2 Booster in ChAdOx1-S-Primed Participants (CombiVacS): A Multicentre, Open-Label, Randomised, Controlled, Phase 2 Trial. Lancet.

[52] Alter G., Yu J., Liu J., Chandrashekar A., Borducchi E.N., Tostanoski L.H., McMahan K., Jacob-Dolan C., Martinez D.R., Chang A. (2021). Immunogenicity of Ad26.COV2.S Vaccine against SARS-CoV-2 Variants in Humans. Nature.

[53] Madhi S.A., Baillie V., Cutland C.L., Voysey M., Koen A.L., Fairlie L., Padayachee S.D., Dheda K., Barnabas S.L., Bhorat Q.E. (2021). Efficacy of the ChAdOx1 NCoV-19 COVID-19 Vaccine against the B.1.351 Variant. N. Engl. J. Med..

[54] Madhi S.A., Kwatra G., Richardson S.I., Koen A.L., Baillie V., Cutland C.L., Fairlie L., Padayachee S.D., Dheda K., Barnabas S.L. (2023). Durability of ChAdOx1 NCoV-19 (AZD1222) Vaccine and Hybrid Humoral Immunity against Variants Including Omicron BA.1 and BA.4 6 Months after Vaccination (COV005): A Post-Hoc Analysis of a Randomised, Phase 1b-2a Trial. Lancet Infect. Dis..

[55] El Sahly H.M., Baden L.R., Essink B., Montefiori D., McDermont A., Rupp R., Lewis M., Swaminathan S., Griffin C., Fragoso V. (2022). Humoral Immunogenicity of the MRNA-1273 Vaccine in the Phase 3 Coronavirus Efficacy (COVE) Trial. J. Infect. Dis..

[56] Benkeser D., Montefiori D.C., McDermott A.B., Fong Y., Janes H.E., Deng W., Zhou H., Houchens C.R., Martins K., Jayashankar L. (2023). Comparing Antibody Assays as Correlates of Protection against COVID-19 in the COVE MRNA-1273 Vaccine Efficacy Trial. Sci. Transl. Med..

[57] Fournier C., Duverlie G., François C., Schnuriger A., Dedeurwaerder S., Brochot E., Capron D., Wychowski C., Thibault V., Castelain S. (2007). A Focus Reduction Neutralization Assay for Hepatitis C Virus Neutralizing Antibodies. Virol. J..

[58] Suntronwong N., Assawakosri S., Kanokudom S., Yorsaeng R., Auphimai C., Thongmee T., Vichaiwattana P., Duangchinda T., Chantima W., Pakchotanon P. (2022). Strong Correlations between the Binding Antibodies against Wild-Type and Neutralizing Antibodies against Omicron BA.1 and BA.2 Variants of SARS-CoV-2 in Individuals Following Booster (Third-Dose) Vaccination. Diagnostics.

[59] Annen K., Morrison T.E., DomBourian M.G., McCarthy M.K., Huey L., Merkel P.A., Andersen G., Schwartz E., Knight V. (2021). Presence and Short-Term Persistence of SARS-CoV-2 Neutralizing Antibodies in COVID-19 Convalescent Plasma Donors. Transfusion.

[60] Whiteman M.C., Bogardus L., Giacone D.G., Rubinstein L.J., Antonello J.M., Sun D., Daijogo S., Gurney K.B. (2018). Virus Reduction Neutralization Test: A Single-Cell Imaging High-Throughput Virus Neutralization Assay for Dengue. Am. J. Trop. Med. Hyg..

[61] Sancilio A.E., D’Aquila R.T., McNally E.M., Velez M.P., Ison M.G., Demonbreun A.R., McDade T.W. (2021). A Surrogate Virus Neutralization Test to Quantify Antibody-Mediated Inhibition of SARS-CoV-2 in Finger Stick Dried Blood Spot Samples. Sci. Rep..

[62] Tan C.W., Chia W.N., Qin X., Liu P., Chen M.I.C., Tiu C., Hu Z., Chen V.C.W., Young B.E., Sia W.R. (2020). A SARS-CoV-2 Surrogate Virus Neutralization Test Based on Antibody-Mediated Blockage of ACE2–Spike Protein–Protein Interaction. Nat. Biotechnol..

[63] Sholukh A.M., Fiore-Gartland A., Ford E.S., Miner M.D., Hou Y.J., Tse L.V., Kaiser H., Zhu H., Lu J., Madarampalli B. (2021). Evaluation of Cell-Based and Surrogate SARS-CoV-2 Neutralization Assays. J. Clin. Microbiol..

[64] Wisnewski A.V., Liu J., Lucas C., Klein J., Iwasaki A., Cantley L., Fazen L., Luna J.C., Slade M., Redlich C.A. (2022). Development and Utilization of a Surrogate SARS-CoV-2 Viral Neutralization Assay to Assess MRNA Vaccine Responses. PLoS ONE.

[65] Abe K.T., Li Z., Samson R., Samavarchi-Tehrani P., Valcourt E.J., Wood H., Budylowski P., Dupuis A.P., Girardin R.C., Rathod B. (2020). A Simple Protein-Based Surrogate Neutralization Assay for SARS-CoV-2. JCI Insight.

[66] Corti D., Purcell L.A., Snell G., Veesler D. (2021). Tackling COVID-19 with Neutralizing Monoclonal Antibodies. Cell.

[67] Wang J.J., Zhang N., Richardson S.A., Wu J.V. (2021). Rapid Lateral Flow Tests for the Detection of SARS-CoV-2 Neutralizing Antibodies. Expert. Rev. Mol. Diagn..

[68] Bian L., Li Z., He A., Wu B., Yang H., Wu Y., Hu F., Lin G., Zhang D. (2022). Ultrabright Nanoparticle-Labeled Lateral Flow Immunoassay for Detection of Anti-SARS-CoV-2 Neutralizing Antibodies in Human Serum. Biomaterials.

[69] Dong T., Han C., Jiang M., Zhang T., Kang Q., Wang P., Zhou F. (2022). A Four-Channel Surface Plasmon Resonance Sensor Functionalized Online for Simultaneous Detections of Anti-SARS-CoV-2 Antibody, Free Viral Particles, and Neutralized Viral Particles. ACS Sens..

[70] Batool R., Soler M., Colavita F., Fabeni L., Matusali G., Lechuga L.M. (2023). Biomimetic Nanoplasmonic Sensor for Rapid Evaluation of Neutralizing SARS-CoV-2 Monoclonal Antibodies as Antiviral Therapy. Biosens. Bioelectron..

[71] Schultz J.S., McCarthy M.K., Rester C., Sabourin K.R., Annen K., DomBourian M., Eisenmesser E., Frazer-Abel A., Knight V., Jaenisch T. (2021). Development and Validation of a Multiplex Microsphere Immunoassay Using Dried Blood Spots for SARS-CoV-2 Seroprevalence: Application in First Responders in Colorado, USA. J. Clin. Microbiol..

[72] Liang Z., Peng T., Jiao X., Zhao Y., Xie J., Jiang Y., Meng B., Fang X., Yu X., Dai X. (2022). Latex Microsphere-Based Bicolor Immunochromatography for Qualitative Detection of Neutralizing Antibody against SARS-CoV-2. Biosensors.

[73] Muruato A.E., Fontes-Garfias C.R., Ren P., Garcia-Blanco M.A., Menachery V.D., Xie X., Shi P.Y. (2020). A High-Throughput Neutralizing Antibody Assay for COVID-19 Diagnosis and Vaccine Evaluation. Nat. Commun..

[74] Mravinacova S., Jönsson M., Christ W., Klingström J., Yousef J., Hellström C., Hedhammar M., Havervall S., Thålin C., Pin E. (2022). A Cell-Free High Throughput Assay for Assessment of SARS-CoV-2 Neutralizing Antibodies. New Biotechnol..

[75] Huang X., Kon E., Han X., Zhang X., Kong N., Mitchell M.J., Peer D., Tao W. (2022). Nanotechnology-Based Strategies against SARS-CoV-2 Variants. Nat. Nanotechnol..

[76] Danh K., Karp D.G., Singhal M., Tankasala A., Gebhart D., de Jesus Cortez F., Tandel D., Robinson P.V., Seftel D., Stone M. (2022). Detection of Neutralizing Antibodies against Multiple SARS-CoV-2 Strains in Dried Blood Spots Using Cell-Free PCR. Nat. Commun..

[77] Nunez F.A., Castro A.C.H., de Oliveira V.L., Lima A.C., Oliveira J.R., de Medeiros G.X., Sasahara G.L., Santos K.S., Lanfredi A.J.C., Alves W.A. (2023). Electrochemical Immunosensors Based on Zinc Oxide Nanorods for Detection of Antibodies Against SARS-CoV-2 Spike Protein in Convalescent and Vaccinated Individuals. ACS Biomater. Sci. Eng..

[78] Tani H., Kimura M., Tan L., Yoshida Y., Ozawa T., Kishi H., Fukushi S., Saijo M., Sano K., Suzuki T. (2021). Evaluation of SARS-CoV-2 Neutralizing Antibodies Using a Vesicular Stomatitis Virus Possessing SARS-CoV-2 Spike Protein. Virol. J..

[79] Carter L.J., Garner L.V., Smoot J.W., Li Y., Zhou Q., Saveson C.J., Sasso J.M., Gregg A.C., Soares D.J., Beskid T.R. (2020). Assay Techniques and Test Development for COVID-19 Diagnosis. ACS Cent. Sci..

[80] Jeong B.S., Cha J.S., Hwang I., Kim U., Adolf-Bryfogle J., Coventry B., Cho H.S., Kim K.D., Oh B.H. (2022). Computational Design of a Neutralizing Antibody with Picomolar Binding Affinity for All Concerning SARS-CoV-2 Variants. Mabs.

[81] Gattinger P., Niespodziana K., Stiasny K., Sahanic S., Tulaeva I., Borochova K., Dorofeeva Y., Schlederer T., Sonnweber T., Hofer G. (2022). Neutralization of SARS-CoV-2 Requires Antibodies against Conformational Receptor-Binding Domain Epitopes. Allergy.

